# Doctor and Patient

**Published:** 1921-07-09

**Authors:** 


### LETTERS OF A LAYMAN.

### Doctor and Patient.

Most people concerned in earning their living, professionally or otherwise, manage to find out how they stand in the estimate of their employers. This probably applies most of all to the journalist. The fierce light that beats round a throne is nothing to that which illuminates the Editor. And, on the whole, the effect is excellent. The truth may be unpalatable, and very often the criticism may be both unfair and malicious. Nevertheless, it is well to know.

### Professional Aloofness.

I have often thought that the man who knows least about the real opinions of his employers—otherwise his patients—is the man of medicine. Possibly he may cultivate aloofness, and possibly aloofness may be thrust upon him. In the general run of cases he is a man of mystery. He gives scant information, writes a prescription, or sends round a mixture, and promises to call again. The patient worries through, and the matter ends. If the man sends for the same doctor next time that he is *malade*, there is presumptive evidence that the patient approved his treatment. At the same time it is not convincing evidence. It may be a case of "Hobson's choice," or that the devil you know is better than the devil you don't know.

Personally, I think that it is in the doctor's interest that he should know something more of lay estimate, even if it is inaccurate. Such knowledge would be an asset in his professional equipment, and it would also tend towards producing greater confidence amongst the general public who often pursue after quacks and well-advertised "curealls" because they lack this confidence. If doctor, and quack, and bottle are all mysteries, well, who will not try an occasional gamble?

### Doctors and Policemen.

It so happens that my own doctor is a man in whose judgment I have implicit confidence. My only complaint is that he is getting old and gradually giving up his practice. I mention this lest it might be said that I am a crank, or the victim of an imaginary grievance.

Let this pass. I have a complaint against doctors in general which I desire to express. If I may be pardoned for the comparison, I regard the doctor's business as being parallel with that of a policeman. Both of them do one part well, and the other they neglect, possibly deliberately, possibly not. A policeman's code prescribes that his duty consists of the prevention and detection of crime—*vide* Sir Howard Vincent and other authorities. But how many of them try to prevent crime? How many of them warn the potential delinquent? An exceedingly small percentage. They prefer to lie in ambush until he succumbs to temptation, and then they place the gyves upon his wrists. He probably realises that this is the surest road to promotion, and that prevention cases do not count. Similarly with the doctor. He tries his best, no doubt, to cure a patient when he is sick. But he makes no effort, nor does he consider it a part of his duty to keep him in good health when he is well. This, I submit, is a vastly more important matter.

Of course it will be argued that this would not butter the doctor's parsnips. I do not agree. The public require only educating in order to realise that even from the expense point of view alone it is cheaper to prevent disease than to cure it when it arrives. Fathers and mothers quack and tinker with their children's health when there is a trifling derangement, and often make matters worse. They shrink from calling in a doctor lest he might regard them as alarmists and smile at their folly. Moreover, as men and women grow old, they become liable to ailments which an altered *régime* might avert. Doctors themselves can furnish hundreds of instances of preventive death caused by the simple process of neglect. The patient did not know, and the doctor did not know.

### Propaganda.

The fault and the remedy lie with the doctor—not with the public. I hold that in a well-ordered home a doctor should examine every member of the household at frequent periods, say once a month, or certainly once a quarter, and go into matters of diet, early rising, and so forth. But this will never come about unless at the instance of the profession. They should, by process of propaganda, demonstrate to the public that as a profession their first duty is the prevention of disease. The public are lamentably ignorant, careless, and apathetic. They need educating. In a matter of this kind they cannot educate themselves. If enlightenment comes it must come from the doctor. It seems to me that a paper like The Hospital might find a mission of this kind its peculiar function. The daily press has too many diverse concerns, and the professional press is not read by the layman. The Hospital ought to be the home journal of practical hygiene. I think it was Dr. Oliver Wendell Holmes who said that at the age of forty every man ought to be a doctor, if he is not a fool—or words to this effect. At all events every man ought to realise that health is the greatest thing in the economy of human affairs, and that health can be conserved. He ought to realise that although the science of medicine will always be progressive there is at present a vast accumulation of scientific knowledge which ought to be at his disposal, not alone when he is sick, but when he is well. So far as I am aware there is at present no means of convincing the public of the importance of constant watchfulness. The worldly prosperity of the family receives due, and sometimes overdue, consideration. But the vital spark on which everything depends is consigned to the region of happy-go-lucky.

IERNE.

